# Impairment of novelty-dependent hippocampal behavioural tagging in *Septin5*-deficient mice

**DOI:** 10.1186/s13041-026-01276-4

**Published:** 2026-02-03

**Authors:** Natsumi Ageta-Ishihara, Naoto Fukumasu, Kazuki Fujii, Yumie Koshidaka, Kenji Tanigaki, Takeshi Hiramoto, Gina Kang, Noboru Hiroi, Tsuyoshi Miyakawa, Keizo Takao, Makoto Kinoshita

**Affiliations:** 1 Department of Biomolecular Science, Faculty of Science, Toho University, 2-2-1 Miyama, Funabashi, Chiba 274-8510 Japan; 2 Department of Molecular Biology, Division of Biological Sciences, Nagoya University Graduate School of Science, Nagoya, Japan; 3 Department of Behavioral Physiology, Faculty of Medicine, University of Toyama, Toyama, Japan; 4 Life Science Research Center, University of Toyama, Toyama, Japan; 5 Research Institute, Shiga Medical Center, Shiga, Japan; 6 Clinical Research Center, Shiga General Hospital, Shiga, Japan; 7 Department of Pharmacology, University of Texas Health Science Center at San Antonio, San Antonio, USA; 8 Department of Cellular and Integrative Physiology, University of Texas Health Science Center at San Antonio, San Antonio, USA; 9 Department of Cell Systems and Anatomy, University of Texas Health Science Center at San Antonio, San Antonio, USA; 10 Division of Systems Medical Science, Center for Medical Science, Fujita Health University, Aichi, Japan; 11 Center for Genetic Analysis of Behavior, National Institute for Physiological Sciences, Aichi, Japan

**Keywords:** Septins, Septin-5, Hippocampus, Behavioural tagging

## Abstract

Septin-5 is a GTP-binding protein implicated in synaptic vesicle exocytosis and 22q11.2 deletion–related neuropsychiatric disorders. We recently showed that *Septin5*-deficient (*Septin5*^−/−^) mice display intact hippocampal spine ultrastructure, but marked deficits in both recent and remote contextual fear memory, whereas cued fear memory is preserved. Building on these findings, we asked whether Septin-5 is required for baseline forms of hippocampus-dependent spatial and object recognition memories, or more selectively for novelty-dependent memory stabilization. Using congenic *Septin5*^−/−^ mice, we performed a behavioural test battery including hippocampus-dependent spatial and object recognition tasks. *Septin5*^−/−^ mice showed normal performance in T-maze (spontaneous and forced alternation), Barnes maze (acquisition and recent/remote spatial reference memory), and object location memory. After 5-min training in the novel object recognition task, short-term recognition memory was indistinguishable between genotypes. Together with our previous report that long-term object recognition after 15-min training is intact in *Septin5*^−/−^ mice, these results indicate that Septin-5 is dispensable for a broad set of hippocampus-dependent spatial and object recognition memories despite contextual fear deficits. In contrast, *Septin5*^−/−^ mice exhibited a selective deficit in behavioural tagging: in wild-type mice, novelty exploration 30 min after 5-min object training converted an otherwise labile trace into a 24-h memory, whereas this novelty-induced stabilization was absent in *Septin5*^−/−^ mice. Thus, Septin-5 is not required for baseline performance in hippocampus-dependent spatial and object recognition tasks, but is implicated in novelty-dependent stabilization of weak hippocampal memories under the established 10-min novelty exposure condition, consistent with a contribution to synaptic tagging–like processes.

**Supplementary Information:**

The online version contains supplementary material available at 10.1186/s13041-026-01276-4.

## Main text

Septins are GTP-binding proteins that form filamentous complexes and contribute to vesicle trafficking and neuronal morphology [1, 2]. Septin-5 is enriched at presynaptic terminals, where it interacts with the SNARE (soluble *N*-ethylmaleimide-sensitive fusion protein attachment protein receptor) machinery and modulates synaptic vesicle exocytosis [3, 4]. Septin-5 is located within the 22q11.2 microdeletion region, a well-established genetic risk locus for schizophrenia and autism spectrum disorder, and mouse studies have implicated *Septin5* gene dosage in the hippocampus and amygdala as a determinant of affiliative social interaction [5].

In our recent study, *Septin5*^−/−^ mice showed preserved hippocampal spine ultrastructure but exhibited marked impairments in both recent and remote contextual fear memory, whereas cued fear conditioning and long-term novel object recognition after strong training remained intact [6]. These findings suggest that Septin-5 contributes to hippocampus-dependent memory processes, but they do not distinguish whether Septin-5 is broadly required for hippocampal learning or preferentially involved in specific forms of memory stabilization.

Behavioural tagging provides a behavioural analogue of the synaptic tagging and capture framework, in which weak hippocampus-dependent learning can be transformed into long-term memory when it is paired, within a restricted time window, with exposure to a novel experience [7]. In the hippocampal novel object recognition paradigm, weak training alone does not support 24-h retention, whereas subsequent exploration of a novel context stabilizes the otherwise labile memory trace [8]. Here, building on this work, we extended our analysis in male congenic *Septin5*^−/−^ with C57BL/6N background mice to a broader battery of hippocampus-dependent spatial and object recognition tasks, and tested whether Septin-5 is broadly required for such baseline memories or is more selectively involved in novelty-dependent behavioural tagging.

We first confirmed that *Septin5*^−/−^ mice do not show general health or motor abnormalities that could confound behavioural assessment. Body weight, rectal temperature, wire-hang latency, and forelimb grip strength were comparable between genotypes, as were spontaneous locomotor activity in the home cage, open-field locomotor activity (consistent with a previous characterization of congenic *Septin5*^−/−^ mice with C57BL/6J background [5]), rota-rod performance, and balance beam performance (Figs. S1–S3). Thus, basic neuromuscular function and activity levels appeared normal in *Septin5*^−/−^ mice under our conditions.

**Fig. 1:**
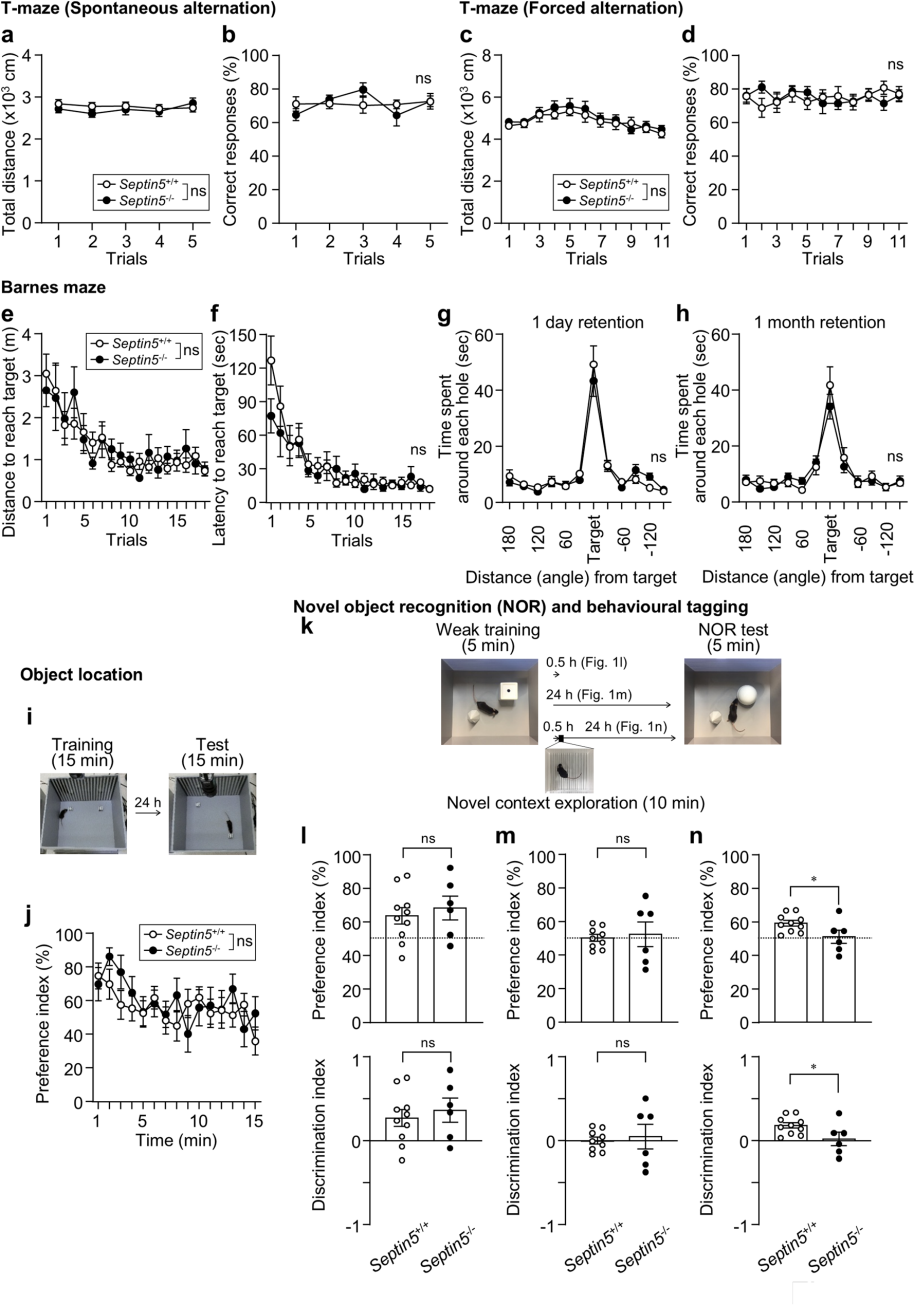
Septin-5 deficiency preserves hippocampus-dependent spatial and object recognition memory but impairs behavioural tagging. **a**, **b** T-maze spontaneous alternation test. **a** Total distance travelled during each trial [genotype main effect, F_1,31_ = 0.43, *p* = 0.52, genotype × trial interaction, F_4,124_ = 0.74, *p* = 0.57]. **b** Percentage of correct responses across trials [genotype main effect, F_1,31_ = 0.0030, *p* = 0.96, genotype × trial interaction, F_4,124_ = 1.39, *p* = 0.24]. *n* = 20 (*Septin5*^+/+^) and 13 (*Septin5*^−/−^), 15–22-week-old male mice; mixed-effects model (REML) with trial as a within-subject repeated factor (subject = mouse) and fixed effects of genotype, trial, and genotype × trial. **c**, **d** T-maze forced alternation test. **c** Total distance travelled during each trial [genotype main effect, F_1,27_ = 0.27, *p* = 0.60, genotype × trial interaction, F_10,270_ = 0.55, *p* = 0.85]. **d** Percentage of correct responses across trials [genotype main effect, F_1,27_ = 0.011, *p* = 0.92, genotype × trial interaction, F_10,270_ = 0.90, *p* = 0.54]. *n* = 14 (*Septin5*^+/+^) and 15 (*Septin5*^−/−^), 12-week-old male mice; mixed-effects model (REML) with trial as a within-subject repeated factor (subject = mouse) and fixed effects of genotype, trial, and genotype × trial. **e–h** Barnes maze. **e** Distance to reach the target hole across training trials [genotype main effect, F_1,30_ = 0.051, *p* = 0.82, genotype × trial interaction, F_17,510_ = 0.54, *p* = 0.93]. **f**, Latency to reach the target hole across training trials [genotype main effect, F_1,30_ = 0.51, *p* = 0.48, genotype × trial interaction, F_17,510_ = 1.17, *p* = 0.29]. *n* = 19 (*Septin5*^+/+^) and 13 (*Septin5*^−/−^), 16–23-week-old male mice; mixed-effects model (REML) with trial as a within-subject repeated factor (subject = mouse) and fixed effects of genotype, trial, and genotype × trial. **g**, **h** Time spent around each hole at the 1-day (**g**) or 1 month (**h**) after training [1 day, genotype main effect, F_1,372_ = 0.29, *p* = 0.59, genotype × angle interaction, F_11,372_ = 0.63, *p* = 0.80; 1 month, genotype main effect, F_1,372_ = 0.32, *p* = 0.57, genotype × angle interaction, F_11,372_ = 0.65, *p* = 0.78]. *n* = 20 (*Septin5*^+/+^) and 13 (*Septin5*^−/−^), 17–24-week-old (**g**) or 22–29-week-old (**h**) male mice; mixed-effects model (REML) with angle as a within-subject repeated factor and fixed effects of genotype, angle, and angle × genotype. **i**, **j** Object location test. **i** Schematic of object location test. **j** Time course of the preference index for the object in the novel location [genotype main effect, F_1,30_ = 0.39, *p* = 0.54, genotype × time interaction, F_14,420_ = 1.16, *p* = 0.31]. *n* = 19 (*Septin5*^+/+^) and 13 (*Septin5*^−/−^), 27–34-week-old male mice; mixed-effects model (REML) with time as a within-subject repeated factor (subject = mouse) and fixed effects of genotype, time, and genotype × time. **k–n** Novel object recognition (NOR) test and behavioural tagging. **k** Schematic of NOR test and behavioural tagging. **l–n** Preference index (upper panels), calculated as 100 × [exploration time of the novel object] / [sum of exploration times for the novel and familiar objects]; the dashed line indicates chance level (50%). Discrimination index (lower panels), calculated as ([exploration time of the novel object] − [exploration time of the familiar object]) / ([exploration time of the novel object] + [exploration time of the familiar object]); 0 indicates no preference (chance level). These indices were calculated from object exploration times measured 0.5 h after short (5-min) training (short-term memory; **l**), 24 h after 5-min training alone (**m**), or 24 h after 5-min training followed 0.5 h later by exposure to a novel context (behavioural tagging; **n**). *n* = 10 (*Septin5*^+/+^) and *n* = 6 (*Septin5*^−/−^) (**l**,** n**), 11–22-week-old male mice, *n* = 9 (*Septin5*^+/+^) and *n* = 6 (*Septin5*^−/−^) (**m**); two-tailed unpaired *t* test (**l**,** n**); Welch’s *t* test (**m**). Data are mean ± SEM; ns, not significant, *p < 0.05.

We then examined hippocampus-dependent spatial and recognition memory. In the T-maze spontaneous and forced alternation tasks, total distance travelled and the percentage of correct responses across trials did not differ between *Septin5*^+/+^ and *Septin5*^−/−^ mice (Fig. 1a–d). Notably, the lack of a genotype effect in the forced (rewarded) alternation task is broadly in line with our previous report in *Septin5*^−/−^ mice on mixed and 129-enriched backgrounds [9]. In the Barnes maze, both genotypes showed similar decreases in distance and latency to reach the target hole during training, and indistinguishable spatial bias toward the target location in probe tests conducted 1 day and 1 month after training (Fig. 1e–h). In the object location task, the time course of preference for the object in the novel location was also comparable between genotypes (Fig. 1i, j). Moreover, congenic *Septin5*^−/−^ mice with C57BL/6 J background showed a normal exploratory response to a single novel object across two 5-min sessions separated by a 30-min interval [5]. Together with our previous finding that long-term novel object recognition after strong (15-min) training is preserved in *Septin5*^−/−^ mice [6], these data indicate that baseline hippocampus-dependent spatial and object recognition memories are broadly intact in the absence of Septin-5.

Next, we asked whether Septin-5 is more selectively involved in novelty-dependent behavioural tagging (Fig. 1k–n). We adopted an established mouse behavioural tagging protocol (5-min training→0.5-h interval→10-min novel context exploration→24-h interval→5-min test) [10]. After short (5-min) training in the novel object recognition task, short-term object memory tested 0.5 h later was apparent in both genotypes, as indicated by preference index (%) values above the chance level (50%) and the discrimination index (DI; chance level = 0) (Fig. 1l); the DI was significantly greater than 0 in *Septin5*^+/+^ mice (one-sample *t* test, *p* = 0.022) and showed a trend in *Septin5*^−/−^ mice (*p* = 0.051), and no significant genotype differences were observed in either the preference index or DI (Fig. 1l). In contrast, 5-min training alone did not support 24-h retention in either genotype; the DI was not significantly greater than 0 in *Septin5*^+/+^ mice (*p* = 0.96) or *Septin5*^−/−^ mice (*p* = 0.76), and no significant differences between genotypes were observed in either index (Fig. 1m). However, when 5-min training was followed 30 min later by novel context exploration, *Septin5*^+/+^ mice showed a clear 24-h preference for the novel object (Fig. 1n), with a DI significantly greater than 0 (one-sample *t* test, *p* = 0.00042). In contrast, *Septin5*^−/−^ mice did not differ from chance (*p* = 0.79). In addition, *Septin5*^−/−^ mice showed lower DI (and the corresponding preference index) than in *Septin5*^+/+^ mice (unpaired two-tailed* t* test, *p* = 0.047) (Fig. 1n); Cohen’s d = 1.12 (95% CI, 0.01–2.20) for DI. Moreover, in *Septin5*^+/+^ mice, the 24-h DI was significantly higher after 5-min training followed by novel context exploration (Fig. 1n) than after 5-min training alone (Fig. 1m) (unpaired two-tailed *t* test, *p* = 0.0035), whereas this difference was not detected in *Septin5*^−/−^ mice (*p* = 0.88). These results support a model in which Septin-5 is dispensable for baseline performance in hippocampus-dependent tasks but appears to be required for novelty-dependent stabilization in the behavioural tagging paradigm tested here.

Together with our previous report that *Septin5* deletion selectively impairs contextual, but not cued, fear memory without overt hippocampal spine abnormalities [6], these findings argue against a global encoding or retrieval deficit and instead point to a more specific role of Septin-5 in processes akin to synaptic or behavioural tagging. Because the novel context exploration session was not video-recorded and exploration was therefore not quantified, we cannot retrospectively assess genotype differences in novelty engagement in this dataset. We also did not directly measure novelty-triggered de novo protein synthesis or molecular signatures of synaptic tagging/capture after novel context exploration in either genotype; therefore, we cannot conclude whether novelty-induced protein synthesis per se is reduced in *Septin5*^−/−^ mice. Given that Septin-5 is enriched at presynaptic terminals and modulates synaptic vesicle exocytosis via interactions with the SNARE machinery [3, 4], one plausible possibility is that *Septin5* deletion alters presynaptic release dynamics and/or hippocampal circuit activation during novelty exposure, thereby failing to sufficiently engage neuromodulator-dependent cascades that normally support novelty-dependent stabilization of weak memory traces [7, 11–14]. In tagging frameworks, weak hippocampus-dependent learning is thought to set a transient tag that can capture plasticity-related proteins induced by a subsequent novel/salient experience within a restricted time window, supported by the ventral tegmental area and locus coeruleus-derived neuromodulatory inputs to the hippocampus [7, 11–14]. Future studies will quantify novelty exploration and test whether increasing novelty exposure duration (e.g., 30 min) can compensate for *Septin5* deletion. Because changing novelty exposure duration constitutes a protocol modification, behavioural tagging should first be re-established under the modified conditions in *Septin5*^+/+^ controls. In addition, it will be important to test whether novelty exposure modulates other hippocampus-dependent memories impaired in *Septin5*^−/−^ mice, such as contextual fear memory [6], building on prior juvenile mouse work supporting a behavioural tagging–like novelty effect on contextual fear memory [15] and validating the protocol under our experimental conditions before assessing genotype-specific effects. Consistent with this view, our recent work on Septin-3 demonstrated that, in dentate granule cell spines, L-LTP induces recruitment of smooth endoplasmic reticulum, leading to larger Ca^2^⁺ responses to synaptic input, and that *Septin3*^−/−^ mice are selectively impaired in long-term, but not short-term, spatial and object memory [16, 17]. Septin-5–dependent synaptic tagging and Septin-3–dependent postsynaptic sER remodeling may thus represent septin-mediated mechanisms that sustain hippocampal memory persistence.

Methods are described in the Supplementary Materials.

## Supplementary Information

Below is the link to the electronic supplementary material.

Supplementary Material 1.

## Data Availability

The datasets generated and analyzed during the current study are available from the corresponding author on reasonable request.

## Abbreviations

SNARE: Soluble *N*-ethylmaleimide-sensitive fusion protein attachment protein receptor
DI: Discrimination index

## References

[1] Sirajuddin M, Farkasovsky M, Hauer F, Kuhlmann D, Macara IG, Weyand M, et al. Structural insight into filament formation by mammalian septins. Nature. 2007;449:311–5. 17637674 10.1038/nature06052

[2] Ageta-Ishihara N, Kinoshita M. Developmental and postdevelopmental roles of septins in the brain. Neurosci Res. 2021;170:6–12. 33159992 10.1016/j.neures.2020.08.006

[3] Beites CL, Campbell KA, Trimble WS. The septin Sept5/CDCrel-1 competes with alpha-SNAP for binding to the SNARE complex. Biochem J. 2005;385:347–53. 15355307 10.1042/BJ20041090 PMC1134704

[4] Beites CL, Xie H, Bowser R, Trimble WS. The septin CDCrel-1 binds syntaxin and inhibits exocytosis. Nat Neurosci. 1999;2:434–9. 10321247 10.1038/8100

[5] Harper KM, Hiramoto T, Tanigaki K, Kang G, Suzuki G, Trimble W, et al. Alterations of social interaction through genetic and environmental manipulation of the 22q11.2 gene Sept5 in the mouse brain. Hum Mol Genet. 2012;21:3489–99. 22589251 10.1093/hmg/dds180 PMC3392117

[6] Ageta-Ishihara N, Fukumasu N, Sakakibara K, Fujii K, Koshidaka Y, Katsuragawa S, et al. Septin5 deficiency impairs both recent and remote contextual fear memory. Mol Brain. 2025;18:85. 41225651 10.1186/s13041-025-01260-4 PMC12613764

[7] Moncada D, Viola H. Induction of long-term memory by exposure to novelty requires protein synthesis: evidence for a behavioral tagging. J Neurosci. 2007;27:7476–81. 17626208 10.1523/JNEUROSCI.1083-07.2007 PMC6672624

[8] Nomoto M, Inokuchi K. Behavioral, cellular, and synaptic tagging frameworks. Neurobiol Learn Mem. 2018;153:13–20. 29535042 10.1016/j.nlm.2018.03.010

[9] Suzuki G, Harper KM, Hiramoto T, Sawamura T, Lee M, Kang G, et al. Sept5 deficiency exerts pleiotropic influence on affective behaviors and cognitive functions in mice. Hum Mol Genet. 2009;18:1652–60. 19240081 10.1093/hmg/ddp086 PMC2733818

[10] Nomoto M, Ohkawa N, Nishizono H, Yokose J, Suzuki A, Matsuo M, et al. Cellular tagging as a neural network mechanism for behavioural tagging. Nat Commun. 2016;7:12319. 27477539 10.1038/ncomms12319 PMC4974651

[11] Frey U, Morris RG. Synaptic tagging and long-term potentiation. Nature. 1997;385:533–6. 9020359 10.1038/385533a0

[12] Moncada D. Evidence of VTA and LC control of protein synthesis required for the behavioral tagging process. Neurobiol Learn Mem. 2017;138:226–37. 27291857 10.1016/j.nlm.2016.06.003

[13] Takeuchi T, Duszkiewicz AJ, Sonneborn A, Spooner PA, Yamasaki M, Watanabe M, et al. Locus coeruleus and dopaminergic consolidation of everyday memory. Nature. 2016;537:357–62. 27602521 10.1038/nature19325 PMC5161591

[14] Moncada D, Ballarini F, Martinez MC, Frey JU, Viola H. Identification of transmitter systems and learning tag molecules involved in behavioral tagging during memory formation. Proc Natl Acad Sci U S A. 2011;108:12931–6. 21768371 10.1073/pnas.1104495108 PMC3150922

[15] Chen N, Tsai TC, Hsu KS. Exposure to novelty promotes long-term contextual fear memory formation in juvenile mice: evidence for a behavioral tagging. Mol Neurobiol. 2020;57:3956–68. 32632604 10.1007/s12035-020-02005-1

[16] Ageta-Ishihara N, Fukazawa Y, Arima-Yoshida F, Okuno H, Ishii Y, Takao K, et al. Septin 3 regulates memory and L-LTP-dependent extension of endoplasmic reticulum into spines. Cell Rep. 2025;44:115352. 40023151 10.1016/j.celrep.2025.115352

[17] Ageta-Ishihara N, Mizukami M, Kinoshita I, Asami Y, Nishioka T, Bito H, et al. Phosphorylated septin 3 delocalizes from the spine base and facilitates endoplasmic reticulum extension into spines via myosin-Va. Mol Brain. 2025;18:43. 40375097 10.1186/s13041-025-01215-9 PMC12079886

