# Supplementary material for: Impairment of novelty-dependent hippocampal behavioural tagging in Septin5-deficient mice
Source: Mol Brain. 2026 Feb 3;19:13. doi: 10.1186/s13041-026-01276-4 (PMC12882602; doi:10.1186/s13041-026-01276-4)
Supplement: Supplementary file 1 — Supplementary Material 1. [file 13041_2026_1276_MOESM1_ESM.pdf]

Supplementary Materials

Methods

Animals

All animal experiments were approved by the Institutional Animal Care and Use Committees of Fujita Health University, the National Institute for Physiological Sciences, University of Toyama, and Nagoya University, and were conducted in accordance with the respective institutional guidelines for the care and use of laboratory animals. Mice were maintained under a 12-h light/dark cycle with free access to food and water.

Septin5 knockout (*Septin5*$^{-/-}$) mice used in this study were derived from a previously established *Septin5* mutant line [1]. To minimize potential influences of genetic background [2], this line was first backcrossed onto the C57BL/6J background for more than 10 generations [3] and subsequently backcrossed for more than 10 generations onto C57BL/6N. *Septin5*$^{-/-}$ and wild-type (*Septin5*$^{+/+}$) mice were obtained by intercrossing *Septin5*$^{+/-}$ heterozygous mice or by *in vitro* fertilization using gametes from *Septin5*$^{+/-}$ mice. Only male mice were used, and the age range at testing is specified in each figure legend.

T-maze

T-maze tasks were performed in an automated T-maze apparatus (O'HARA & Co.), essentially as described previously [4, 5]. Briefly, the maze consisted of 8-cm-wide runways with 25-cm-high opaque walls and was divided into six compartments by computer-controlled sliding doors: a start box (S1), a central stem (S2; 13 × 24 cm), two goal arms (A1 and A2; 11.5 × 20.5 cm), and two return alleys (P1 and P2) connecting each arm to the start box. When a mouse approached a door, the next door opened and the previous one closed, allowing the animal to move through the maze without being handled by the experimenter.

For the spontaneous alternation task, mice were placed in the start box (S1) at the beginning of each trial and allowed to choose either arm (A1 or A2). After entering an arm, the doors guided the mouse through the corresponding return alley (P1 or P2) back to S1, which marked the end of a trial. Upon return to S1, the mouse remained in the start box, and the next trial was initiated after a 3-s pause. Thus, trials were conducted in a continuous manner without a discrete inter-trial interval involving removal of the animal from the apparatus. Each session consisted of 10 consecutive trials per day for 5 days (cut-off time, 1800 s per session). An alternation was scored as correct when the mouse entered the arm opposite to that chosen on the immediately preceding trial; re-entry into the same arm was scored as an error. The percentage of correct responses and total distance travelled were calculated from the tracking data.

In the forced alternation task, mice were food-restricted for at least 1 week before the start of training and throughout the task with body weight maintained at approximately 85% of the ad libitum level. After a 30-min habituation session with all doors open, mice were trained in daily sessions of 10 trials for 11 consecutive days. Each trial consisted of a forced-choice run followed by a free-choice run. In the forced-choice run, only one arm (A1 or A2) was made accessible according to a pseudo-random Gellermann sequence; the mouse was required to enter this arm and consume the pellet. When the pellet was eaten or 30 s had elapsed, the doors opened to return the mouse via P1 or P2 to S1. After the mouse returned to S1 following the forced-choice run, it remained in the start box for 3 s before the initiation of the free-choice run, during which both arms were opened. Reward was available only in the arm opposite to that visited during the forced-choice run. Selection of the previously unvisited arm was counted as a correct response; choosing the previously rewarded arm was scored as an error, and the mouse was confined in that arm for 10 s as a penalty. After completion of the free-choice run, the mouse returned to S1 via the return alley, which marked the end of the trial. The next trial was initiated 3 s after the mouse reached S1, during which time the mouse remained in the maze and was not removed from the apparatus. For each session, the percentage of correct choices and the total distance travelled were extracted from the automated tracking data.

Barnes maze

The Barnes maze task was conducted on a circular white platform (diameter, 1 m) with twelve evenly spaced peripheral holes (4 cm in diameter), essentially following our previous procedures [6]. Briefly, an escape box (17 × 13 × 7 cm) filled with bedding was placed beneath one designated target hole, which was assigned pseudo-randomly for each mouse. Several distal visual cues were positioned around the apparatus, and illumination was adjusted so that the overall light intensity on the platform exceeded 1,000 lux (approximately 1,200 lux at the centre). During training, mice were released from the centre of the platform and allowed to search for and enter the escape box, at which point they were removed from the maze. Each mouse underwent three trials per day for six consecutive days. The distance travelled before reaching the target and the latency to enter the escape box were recorded. For probe tests, the escape box was removed and mice were allowed to explore the platform freely for 3 min. Probe sessions were performed 1 day and 1 month after the last training session, and the time spent in the vicinity of each hole was quantified.

Object location

Object location memory was assessed in a square chamber (40 × 40 × 30 cm) in which one wall was covered with vertical stripes as a distal spatial cue. Two identical cube objects (dice with all faces

labeled 6) were used as to-be-remembered landmarks. Before testing, mice were habituated to the empty chamber by allowing free exploration for 10 min on three separate sessions. The object location test was conducted over two consecutive days. On day 1, two identical objects were placed near the corner adjacent to the striped wall, and each mouse was placed in the left-front corner of the chamber and allowed to explore freely for 15 min. On day 2, one object was shifted to the diagonally opposite corner (novel location), while the other remained in the original position (familiar location), and mice were again allowed to explore for 15 min. The time spent in predefined areas surrounding each object was measured. The preference index (%) was calculated as 100 × [exploration time of the novel object] / [sum of exploration times for the novel and familiar objects].

Novel object recognition (NOR) and behavioural tagging

NOR and behavioural tagging were performed using a modified version of previously described procedures [7, 8]. Briefly, the NOR task was conducted in a rectangular acrylic arena (250 × 290 × 290 mm); the front wall was wrapped with white tape, and the remaining walls were made of gray acrylic. Three white ceramic objects were used: a cube (6 × 6 × 6 cm), a sphere (diameter 6 cm, height 7.3 cm), and an octagonal pyramid (diameter 4.5 cm, height 7.4 cm). To habituate the animals to the experimenter and apparatus, mice were handled and allowed to freely explore the empty arena for 6 min per day for 4 consecutive days. For short (5-min) NOR training, two identical objects were placed near opposite corners along the diagonal of the arena, and the mouse was allowed to explore for 5 min. Memory was tested either 0.5 h (short-term memory) or 24 h (long-term memory) after training by replacing one of the familiar objects with a novel object and allowing the mouse to explore for 5 min. Exploration times directed toward each object were measured. The preference index (%) was calculated as 100 × [exploration time of the novel object] / [sum of exploration times for the novel and familiar objects], and the discrimination index was calculated as ([exploration time of the novel object] – [exploration time of the familiar object]) / ([exploration time of the novel object] + [exploration time of the familiar object]).

Behavioural tagging was examined by combining NOR with novel context exploration (NCE). NCE was carried out in a separate rectangular box (175 × 165 × 300 mm) located in the same testing room as the NOR arena, with a transparent front wall and opaque white side and back walls; the floor consisted of 2-mm stainless-steel rods spaced 5 mm apart. Mice first underwent short NOR training (5 min exploration of two identical objects in the NOR arena). They were placed in the NCE apparatus 0.5 h later and allowed to freely explore the novel context for 10 min. Twenty-four h after the NCE, memory for the objects was assessed in the NOR arena by replacing one of the familiar objects with a novel object and allowing 5 min of exploration. Object exploration times were quantified, and the

preference index and discrimination index were calculated as described above.

Wire hang test and grip strength test

Wire hang and grip strength tests were performed according to our previous report [4]. Briefly, the wire hang apparatus consisted of a 21.25 × 22 × 30 cm box with a 10 × 10 cm wire grid mounted at the center of the ceiling that could be rotated to an inverted position. Mice were placed on the upper surface of the grid, and the grid was then inverted so that the animals hung upside down. The latency to fall from the grid was recorded, with a maximal cutoff time of 180 s.

Forelimb grip strength was measured using a grip strength meter equipped with a 4 × 4 cm wire grid attached to a force transducer. Mice were held by the tail and brought close to the grid; once they grasped the grid with both forepaws only, they were gently pulled backward in a horizontal plane until they released the grid, and the peak tensile force (N) was recorded. This procedure was repeated three times in succession for each mouse, and the highest value was used for analysis.

Home cage monitoring

Home-cage monitoring was performed according to our previous methods [6]. Briefly, two male mice of the same genotype that had been reared in separate cages (non–cage mates, body weight difference <10%) were paired and housed together in a standard home cage equipped with a ceiling-mounted video camera. Their positions were continuously recorded for 7 consecutive days, and locomotor activity for each mouse was quantified as the total distance travelled, summed in 1-h bins, using an automated tracking pipeline.

Open field test

Open-field test was performed according to our previous methods [4]. Briefly, mice were placed in the left-front corner of a square open-field chamber (40 × 40 × 30 cm) and allowed to explore freely for 120 min. Locomotor activity was quantified as distance travelled per unit time using an automated tracking system.

Rota-rod test

Rota-rod test was performed following established protocols [6]. Briefly, mice were placed on a 3-cm-diameter rotating rod positioned 15 cm above the floor, which was linearly accelerated from 4 to 40 rpm over 5 min, and the latency to fall was recorded. Each mouse underwent three trials on the first day and three trials on the second day (six trials in total).

Balance beam test

Balance beam test was performed according to previous methods [6]. Briefly, mice were required to traverse a horizontal cylindrical beam (length, 1 m; diameter, 2.7 or 1.1 cm) positioned 50 cm above the floor, with a black goal box attached at the far end. For each traversal, the moving speed and the number of slips were quantified.

Quantification and statistical analysis

Data are presented as means ± SEM (standard error of the mean). Statistical analyses were performed using GraphPad Prism 10 (GraphPad Software). For comparisons between two independent groups, distributional assumptions were first examined with the Shapiro–Wilk normality test. When normality was not rejected ($p > 0.05$), variances were compared with the F test; datasets with equal variances were analyzed with a two-tailed unpaired *t* test, whereas those with unequal variances were analyzed with Welch's t test. When the Shapiro–Wilk test indicated deviation from normality ($p < 0.05$), group differences were evaluated with the Mann–Whitney test. For repeated-measures data with two genotypes, we used mixed-effects models with REML, treating mouse identity as a random effect and including genotype, the within-subject factor (time, trial, or angle, as appropriate), and their interaction as fixed effects. Graphical diagnostics (residual plots, homoscedasticity plots, and QQ plots) were inspected and did not reveal major violations of model assumptions. The statistical test used, sample size (n), p values, and definition of error bars are described in the figure legends.

References

1. Peng XR, Jia Z, Zhang Y, Ware J, Trimble WS: **The septin CDCrel-1 is dispensable for normal development and neurotransmitter release.** *Mol Cell Biol* 2002, **22:**378-387.
2. Hiroi N: **Critical reappraisal of mechanistic links of copy number variants to dimensional constructs of neuropsychiatric disorders in mouse models.** *Psychiatry Clin Neurosci* 2018, **72:**301-321.
3. Harper KM, Hiramoto T, Tanigaki K, Kang G, Suzuki G, Trimble W, Hiroi N: **Alterations of social interaction through genetic and environmental manipulation of the 22q11.2 gene Sept5 in the mouse brain.** *Hum Mol Genet* 2012, **21:**3489-3499.
4. Ageta-Ishihara N, Takao K, Miyakawa T, Kinoshita M: **Comprehensive behavioral phenotyping of male Septin 3-deficient mice reveals task-specific abnormalities.** *Mol Brain* 2025, **18:**71.
5. Shoji H, Hagihara H, Takao K, Hattori S, Miyakawa T: **T-maze forced alternation and left-right discrimination tasks for assessing working and reference memory in mice.** *J Vis Exp* 2012.
6. Ageta-Ishihara N, Yamazaki M, Konno K, Nakayama H, Abe M, Hashimoto K, Nishioka T, Kaibuchi K, Hattori S, Miyakawa T, et al: **A CDC42EP4/septin-based perisynaptic glial scaffold facilitates glutamate clearance.** *Nat Commun* 2015, **6:**10090.
7. Ageta-Ishihara N, Fukumasu N, Sakakibara K, Fujii K, Koshidaka Y, Katsuragawa S, Tanigaki K, Hiramoto T, Kang G, Hiroi N, et al: **Septin5 deficiency impairs both recent and remote contextual fear memory.** *Mol Brain* 2025, **18:**85.
8. Nomoto M, Ohkawa N, Nishizono H, Yokose J, Suzuki A, Matsuo M, Tsujimura S, Takahashi Y, Nagase M, Watabe AM, et al: **Cellular tagging as a neural network mechanism for behavioural tagging.** *Nat Commun* 2016, **7:**12319.

Supplementary Figures

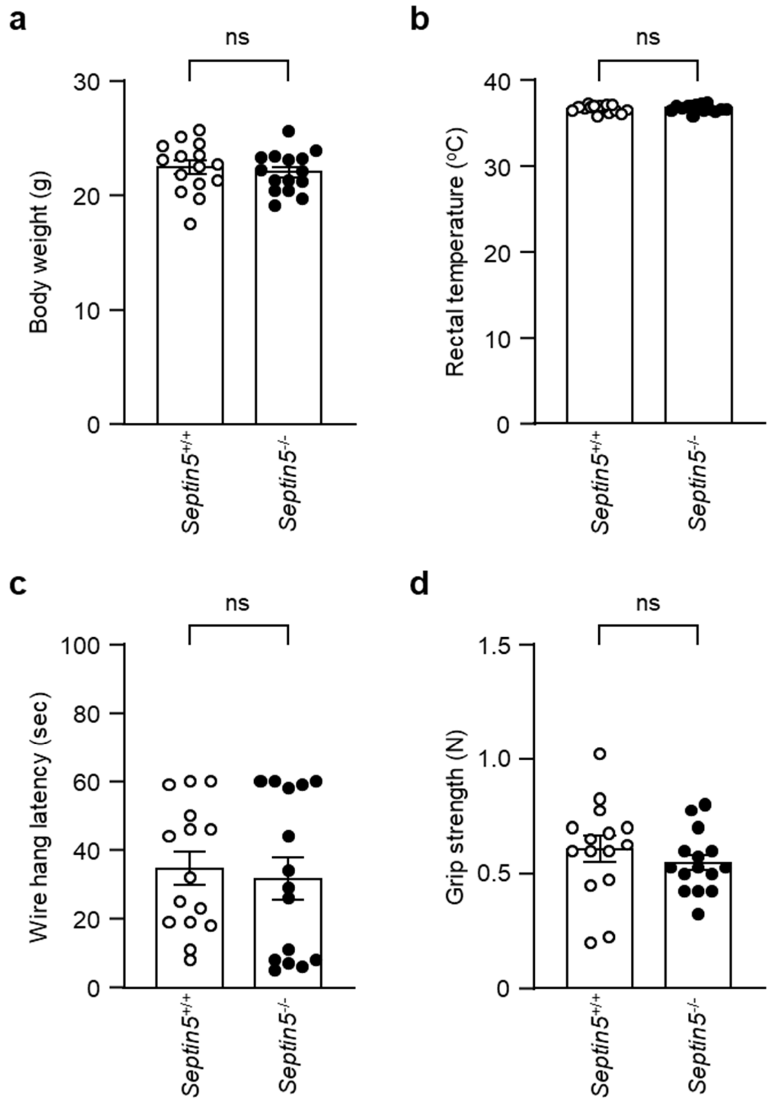

Figure S1

Normal general health and neuromuscular function in *Septin5*$^{-/-}$ mice.

a, Body weight.

b, Rectal temperature.

c, Wire-hang latency.

d, Forelimb grip strength.

n = 15 (*Septin5*$^{+/+}$) and 15 (*Septin5*$^{-/-}$), 7-week-old male mice; two-tailed unpaired *t* test (**a**, **b**, **d**); Mann–Whitney test (**c**). Data are mean ± SEM; ns, not significant.

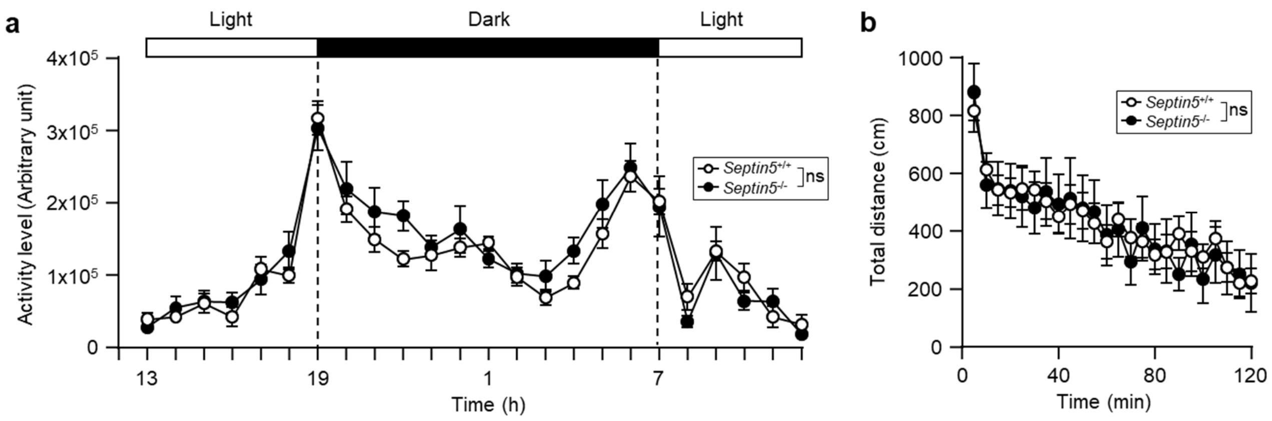

Figure S2

Normal spontaneous locomotor activity and open-field exploration in *Septin5*$^{-/-}$ mice.

a, Mean spontaneous locomotor activity in the home cage [genotype main effect, $F_{1,12} = 0.38$, $p = 0.55$, genotype × time interaction, $F_{23,276} = 1.06$, $p = 0.39$]. n = 7 (*Septin5*$^{+/+}$) and 7 (*Septin5*$^{-/-}$) pairs, 18–19-week-old male mice.

b, Total distance travelled in the open-field test [genotype main effect, $F_{1,31} = 0.0065$, $p = 0.94$, genotype × time interaction, $F_{23,713} = 0.65$, $p = 0.89$]. n = 20 (*Septin5*$^{+/+}$) and 13 (*Septin5*$^{-/-}$), 12–19-week-old male mice.

Mixed-effects model (REML) with time as a within-subject repeated factor (subject = mouse) and fixed effects of genotype, time, and genotype × time. Data are mean ± SEM.

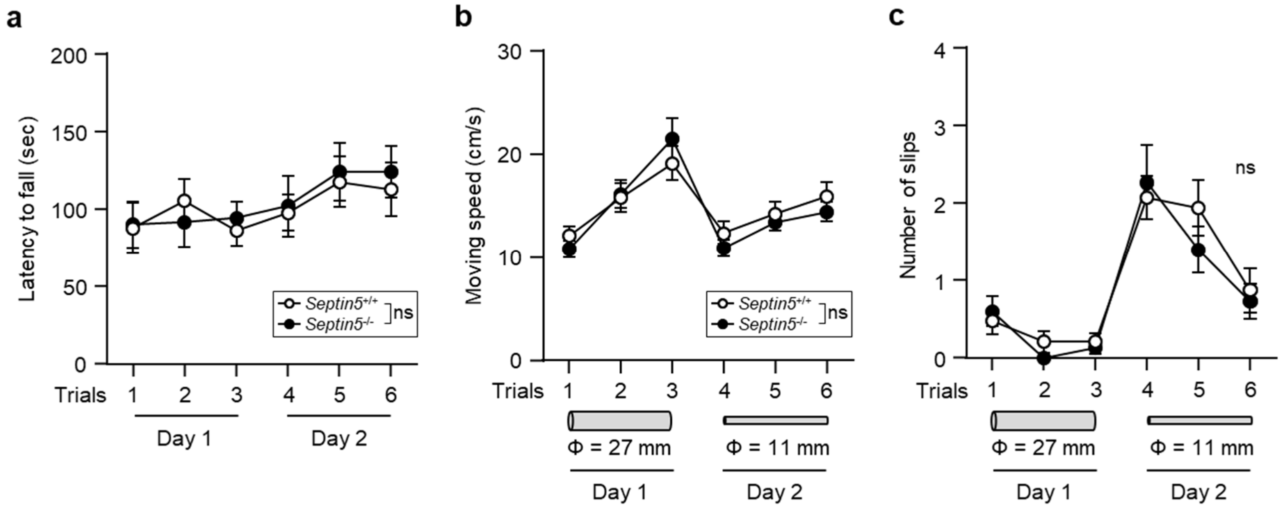

Figure S3

Normal motor coordination in *Septin5*$^{-/-}$ mice.

a, Latency to fall assessed by the rota-rod test [genotype main effect, $F_{1,28} = 0.039$, $p = 0.85$, genotype × trial interaction, $F_{5,140} = 0.30$, $p = 0.91$].

b, **c**, Moving speed (**b**) [genotype main effect, $F_{1,28} = 0.11$, $p = 0.74$, genotype × trial interaction, $F_{5,140} = 1.01$, $p = 0.41$] and number of slips (**c**) [genotype main effect, $F_{1,28} = 0.39$, $p = 0.54$, genotype × trial interaction, $F_{5,140} = 0.55$, $p = 0.73$] in the balance beam test.

n = 15 (*Septin5*$^{+/+}$) and 15 (*Septin5*$^{-/-}$), 8-week-old male mice; mixed-effects model (REML) with trial as a within-subject repeated factor (subject = mouse) and fixed effects of genotype, trial, and genotype × trial. Data are mean ± SEM.
